# Added Value of Clinical Sequencing: WGS-Based Profiling of Pharmacogenes

**DOI:** 10.3390/ijms21072308

**Published:** 2020-03-26

**Authors:** Sylvan M. Caspar, Timo Schneider, Janine Meienberg, Gabor Matyas

**Affiliations:** 1 Center for Cardiovascular Genetics and Gene Diagnostics, Foundation for People with Rare Diseases, 8952 Schlieren-Zurich, Switzerland; caspar@genetikzentrum.ch (S.M.C.); praktikanten@genetikzentrum.ch (T.S.); meienberg@genetikzentrum.ch (J.M.); 2 Laboratory of Translational Nutrition Biology, Department of Health Sciences and Technology, ETH Zurich, 8603 Schwerzenbach, Switzerland; 3 Zurich Center for Integrative Human Physiology, University of Zurich, 8057 Zurich, Switzerland

**Keywords:** *CYP2D6*, DPWG, gnomAD, next-generation sequencing, precision medicine, pharmacogenetics, PGx, whole-genome sequencing

## Abstract

Although several pharmacogenetic (PGx) predispositions affecting drug efficacy and safety are well established, drug selection and dosing as well as clinical trials are often performed in a non-pharmacogenetically-stratified manner, ultimately burdening healthcare systems. Pre-emptive PGx testing offers a solution which is often performed using microarrays or targeted gene panels, testing for common/known PGx variants. However, as an added value, whole-genome sequencing (WGS) could detect not only disease-causing but also pharmacogenetically-relevant variants in a single assay. Here, we present our WGS-based pipeline that extends the genetic testing of Mendelian diseases with PGx profiling, enabling the detection of rare/novel PGx variants as well. From our in-house WGS (PCR-free 60× PE150) data of 547 individuals we extracted PGx variants with drug-dosing recommendations of the Dutch Pharmacogenetics Working Group (DPWG). Furthermore, we explored the landscape of DPWG pharmacogenes in gnomAD and our in-house cohort as well as compared bioinformatic tools for WGS-based structural variant detection in *CYP2D6*. We show that although common/known PGx variants comprise the vast majority of detected DPWG pharmacogene alleles, for better precision medicine, PGx testing should move towards WGS-based approaches. Indeed, WGS-based PGx profiling is not only feasible and future-oriented but also the most comprehensive all-in-one approach without generating significant additional costs.

## 1. Introduction

Pharmacogenetics is primarily concerned with how genetic variation affects individual drug response [1]. In the current genomics era, technological advances allow the unprecedented implementation of pharmacogenetics and its importance is becoming increasingly evident. Less than 10% of drugs reach approval [2]; the costs of drug development, following Eroom’s law, have risen to almost €3 bn per marketed drug [3]; healthcare costs are increasing; and the number of annual deaths and costs due to adverse drug events (ADEs) are estimated to be 197’000 and €79 bn, respectively, in the EU [4]. In the US, the annual cost caused by nonoptimized medical therapy is estimated to be approximately $530 bn [5]. In light of these facts, there is a need for improvement in both drug development and healthcare systems. Pharmacogenetics may provide a solution to tackle these issues in the form of pre-emptive pharmacogenetic (PGx) testing for drug selection and dosing as well as PGx-based stratification of clinical trials. While in oncology pharmacogenetics has already been implemented to identify patients with a higher chance to benefit from a treatment according to tumor-driver variants [6], in non-cancer cases drug selection and dosing as well as clinical trials are often performed in a non-pharmacogenetically-stratified manner.

For pre-emptive PGx testing, a variety of commercially available providers and assays exist, including Abomics (abomics.fi), bio.logis (biologis.de), PharmacoScan (thermofischer.com), Sonogen (sonogen.eu), and VeriDose (agenabio.com) which use PCR- and/or microarray-based techniques. In addition, targeted gene panels using next-generation sequencing (NGS) are available, such as the AmpliSeq Pharmacogenomics Research Panel (thermofisher.com), PGRNseq [7], and the VeraCode ADME Core Panel (illumina.com). The focus mostly lies on screening for the most common and well described (known) PGx variants affecting pharmacokinetics or -dynamics, often referred to as star (*) alleles. For translation of the relevant genotypes to actionable PGx recommendations, guidelines of international experts are frequently invoked, such as the Dutch Pharmacogenetics Working Group (DPWG) [8] or the Clinical Pharmacogenetics Implementation Consortium (CPIC) [1]. As pre-emptive PGx testing is not yet routine in most clinics, several trials are currently evaluating cost-effectiveness and patient outcomes, including (i) the EU-funded PREPARE clinical study [9], in which microarray-based testing is used, enabling easy-to-use and platform-independent decision support by providing results on the Medication Safety Code system [10]; (ii) the CLIPMERGE PGx Program [11]; (iii) the eMERGE-PGx project [12], the latter two deploying PGRNseq [7], enabling the screening of selected pharmacogenes and integrating the results of PGx profiling into electronic health records (EHRs).

NGS-based approaches have the advantage of additionally detecting novel (yet-unknown) and rare but pharmacogenetically-relevant variants, which may contribute to interindividual variability, allowing better PGx profiling and personalized medicine [13]. Indeed, the increasingly used whole-exome sequencing (WES) and especially whole-genome sequencing (WGS) may not only be used for the diagnosis of Mendelian diseases, but also represent an untapped data source for pre-emptive PGx testing due to sequencing all (known) genes to a large extent, including pharmacogenes [14]. However, WGS-based PGx profiling is not yet common and the true contribution of common/known PGx variants compared to rare/novel (and thus usually not tested) variants is unknown. In addition, due to short-read-related alignment ambiguities in repetitive or homologous genomic regions, accurate variant calling in the cytochrome P450 (CYP) enzyme superfamily, members of which are responsible for the biotransformation of ~70%–80% of all drugs, is challenging. The most notable example is the pharmacogene *CYP2D6*, which is highly polymorphic and homologous to its two pseudogenes and metabolizes ~25% of all drugs in clinical use [15]. As in such homologous/repetitive genomic regions, standard short-read-based alignment and variant calling pipelines may not reliably detect all pharmacogenetically-relevant variants, specialized software tools have been introduced to call difficult-to-detect variants, including *CYP2D6* structural variants (SVs) [15,16,17,18]. These specialized short-read-based software tools, however, have not been independently evaluated.

To address these issues, we present our first-of-its-kind pipeline from raw short-read WGS data to diagnosis and PGx profiling, thereby implementing drug-dosing recommendations according to the DPWG guidelines. Furthermore, we assess relative allele frequencies of both common/known PGx and rare/novel loss-of-function (LOF) variants in current DPWG pharmacogenes in the largest publicly available population-based reference dataset gnomAD (v2.1.1 and v3) and in our large in-house WGS cohort as well as evaluate the accuracy of WGS-based *CYP2D6* (structural) variant detection and discuss the future perspectives of PGx implementation.

## 2. Results

### 2.1. Pipeline from Raw Data to Molecular Genetic Diagnosis, PGx Report, and Medication Safety Card (MSC)

**Figure 1:**
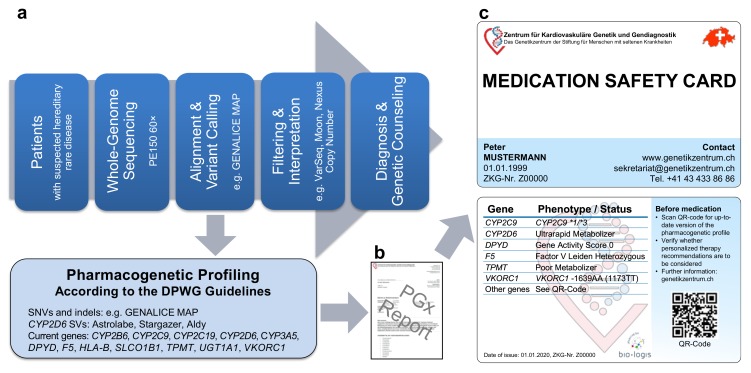
Our whole-genome sequencing (WGS)-based pharmacogenetic (PGx) profiling. (**a**) Pipeline from whole-genome sequencing data to (**b**) PGx report and (**c**) individualized PGx profile in credit card format (Medication Safety Card). Abbreviations: DPWG, Dutch Pharmacogenetic Working Group; indels, insertions/deletions; SNVs, single nucleotide variants; SVs, structural variants.

Our WGS-based clinical sequencing pipeline has been expanded to include PGx profiling (Figure 1a). For this, pharmacogenetically-relevant variants were extracted from WGS data and interpreted according to the quarterly-updated DPWG guidelines. Similar to the PREPARE trial [9,10], our expanded pipeline outputs individualized treatment recommendations on a PGx report (Figure 1b) as well as on a PGx profile (Figure 1c) in credit card format (medication safety card, MSC), both of which can accordingly be used for better patient care. Currently, our pipeline analyzes the most established 45 single nucleotide variants (SNVs) and insertions/deletions (indels) in 11 genes as well as an HLA-B*5701 tagging variant and the deletion/duplication of *CYP2D6*, providing DPWG recommendations for 77+X (X = oral contraceptives) drugs (hereafter referred to as DPWG variants). As additional pharmacogenetically-relevant variants are expected to be described in the future, the MSC contains a QR code, providing access to an appropriate portal (accessible online or via app), containing up-to-date recommendations for personalized drug selection and dosing. Thus, our expanded pipeline not only allows WGS-based diagnosis but also individualized PGx recommendations for drug selection and dosing.

### 2.2. Evaluation of PGx Variants in gnomAD and Our In-House Cohort

**Figure 2:**
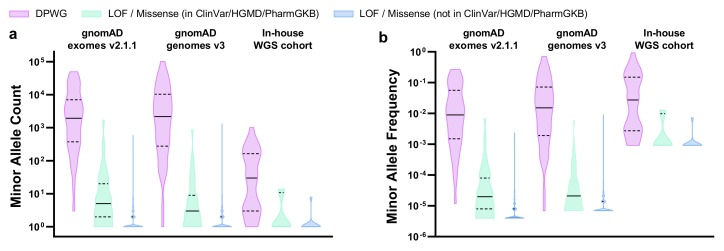
Violin plots showing the distributions of (**a**) minor allele counts and (**b**) minor allele frequencies in 11 DPWG genes (*CYP2B6*, *CYP2C9*, *CYP2C19*, *CYP2D6*, *CYP3A5*, *DPYD*, *F5*, *SLCO1B1*, *TPMT*, *UGT1A1*, *VKORC1)* and an HLA-B*5701 tagging variant in gnomAD exomes v2.1.1, gnomAD genomes v3, and our in-house WGS cohort. Horizontal lines indicate median, horizontal dashed lines indicate quartiles. Plots were generated using Graphpad Prism 8 (Graphpad Software, CA, USA). Abbreviations: DPWG, Dutch Pharmacogenetics Working Group; LOF, loss of function.

We explored the landscape of 11 DPWG pharmacogenes as well as an HLA-B*5701 tagging variant, in gnomAD exomes v2.1.1, gnomAD genomes v3, and our in-house WGS cohort. For this, we calculated the relative allele frequencies not only of DPWG but also of LOF/missense variants that most likely affect the function of pharmacogenes as well. In total, we detected 966, 637, and 51 pharmacogenetically most likely relevant variants in gnomAD exomes v2.1.1, gnomAD genomes v3, and our in-house WGS cohort, respectively (Table 1, Supplementary Table S1). The 45 DPWG variants account for the vast majority (~98%, ranging from 59% to 100%) of detected alleles, depending on gene, dataset, and subpopulation (Supplementary Table S1). Notably, 55%, 51%, and 49% of the DPWG variants occurred with a minor allele frequency (MAF) between 0.1% and 5%, while 25%, 31%, and 38% of the DPWG variants occurred with a MAF above 5% in gnomAD exomes v2.1.1, gnomAD genomes v3, and our in-house cohort, respectively (Table 1, Figure 2). In contrast, 99.8%, 98.4%, and 100.0% of the novel (i.e., not listed in ClinVar, HGMD or PharmGKB) LOF/missense variants can be classified as rare, occurring with a MAF <0.1%. The full table of retained variants is listed in Supplementary Table S1. Furthermore, in our in-house WGS cohort of 547 genomes we detected 37 of the 45 DPWG variants (Table 1), on average, 6 of which occurred per genome (Supplementary Figure S1).

**Figure 3:**
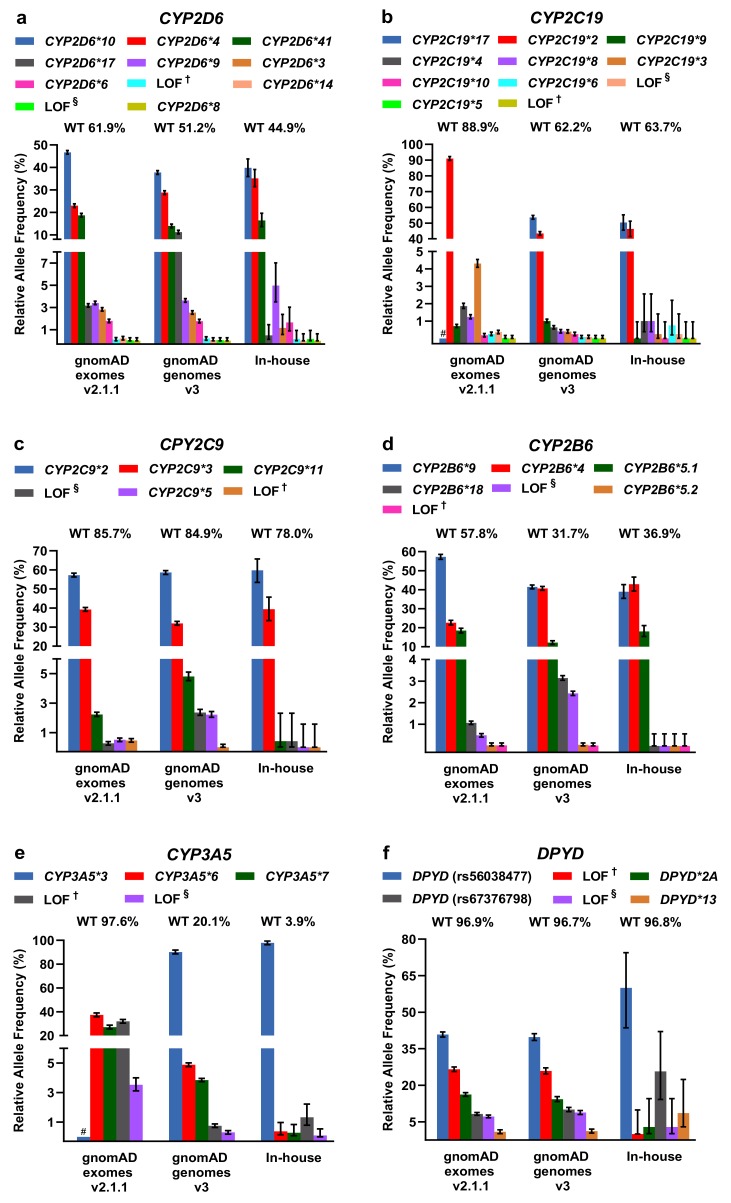

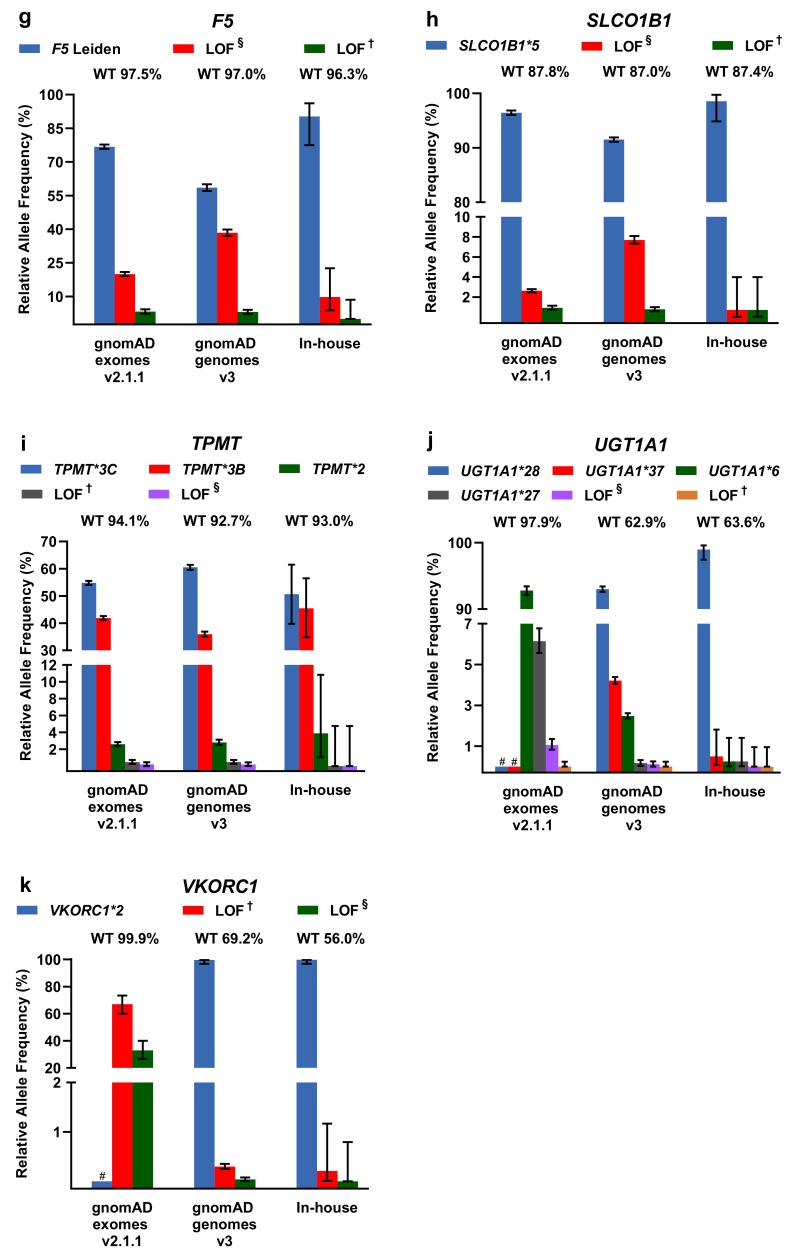
(**a**–**k**) Relative allele frequencies of pharmacogenetically-relevant variants in gnomAD exomes and genomes as well as in our in-house WGS cohort. The percentage of corresponding wildtype (WT) alleles is denoted. Note that wildtype status was inferred under the assumption that the listed variants detected in the same gene occur in *trans* and that no additional pharmacogenetically- relevant variant occurs at the wildtype allele. Note that some of the DPWG variants are not covered in gnomAD exomes (denoted with # in the graphs of CYP2C19*17, CYP3A5*3, UGT1A1*28,*37, and VKORC1*2). Error bars indicate 95% confidence intervals. ^†^ Loss-of-function (LOF) variants listed in HGMD, ClinVar, or PharmGKB. ^§^ LOF variants not listed in HGMD, ClinVar, or PharmGKB in the context of drug response.

For LOF/missense and DPWG variants, including the respective DPWG star (*) alleles, we calculated relative allele frequencies and compared them among the three databases (Figure 3). To infer the number of wildtpye (*1) alleles, we made the assumption that variants detected in one gene occur in *trans*. In general, the inferred wildtype allele is the most prominent allele, however, in the genes *CYP2D6* (in-house genomes), *CYP2B6* (gnomAD and in-house genomes), and *CYP3A5* (gnomAD and in-house genomes), known DPWG variants are more frequent than the inferred wildtype (Figure 3a,d,e). Specifically, the DPWG variants CYP2D6*3,*4,*6,*8,*9,*10,*14,*17,*41 and CYP2B6*4,*5,*,*9*,*18, when combined, amount to more alleles than the respective inferred wildtype alleles CYP2B6*1 and CYP2D6*1. In addition, the CYP3A5*3 allele (NM_000777.4: c.219-237A>G, intronic splicing variant; www.pharmgkb.org/vip/PA166170041) is the most common allele of *CYP3A5*, constituting the majority of alleles in gnomAD genomes and our in-house WGS cohort, whereas it is not captured in WES-based gnomAD exomes. Similarly, the variants CYP2C19*17 (c.-806C>T, promoter), VKORC1*2 (c.174-136C>T, deep intronic), and UGT1A1 *28/*37 (c.-41_-40dup/ c.-43_-40dup) are not present in gnomAD exomes (cf. CYP2C19*17 and CYP3A5*3 are not covered in the assessed in-house WES samples as well). Note that 60× WGS performed better (i.e., resulted in better coverage) than 30× WGS and 100× WES in all investigated categories (Supplementary Figure S2).

Other less common DPWG variants, however, are not present in our primarily Caucasian in-house WGS cohort, likely due to the relatively small cohort size or because variants are predominantly detected in different subpopulations such as the CYP2C9*5 allele in individuals of African descent (Supplementary Table S1). In contrast, the variants CYP2C9*2 and CYP2C9*3 are significantly more frequent in the European subpopulations of gnomAD exomes v2.1.1 and gnomAD genomes v3 compared to the African subpopulation (Supplementary Table S1). Overall, however, the contributions are comparable among the three databases and with previous estimates [13,19].

Moreover, in our in-house cohort of 547 genomes, we screened for DPWG and LOF/missense variants occurring in the same gene. In our cohort, 19 individuals harbor such co-occurring variants, in all but one of which the additional LOF/missense variant may also cause decreased or no enzyme function. One individual harbors not only the allele CYP2C19*17 (c.-806C>T) causing the ultrarapid metabolizer phenotype, but also the allele CYP2C19*2 causing poor metabolizer phenotype as well as the hitherto undescribed *CYP2C19* splicing variant c.643-2A>T likely disrupting gene function (cf. possibly LOF). Although phasing was not possible for these variants, not considering the novel splicing variant could result in a misclassification of the *CYP2C19* metabolizer status. Furthermore, because many of our patients might be treated with an anticoagulant due to their cardiovascular phenotype, we screened for *CYP2C9* and *VKORC1* variants affecting phenprocoumon/warfarin dosing. Thereby, we detected 113 individuals homozygous for the high phenprocoumon/warfarin sensitivity variant VKORC1*2 as well as 11 and 2 individuals homozygous for the poor metabolizer variants CYP2C9*2 and CYP2C9*3, respectively. We also detected 5 individuals homozygous for VKORC1*2 and CYP2C9*2, requiring a reduction of the phenprocoumon/warfarin starting dose to ~35% compared to VKORC1*1/CYP2C9*1 individuals [20].

### 2.3. Comparison of CYP2D6 Calling Tools from WGS Data

Three command-line-based bioinformatic tools, Astrolabe (previously Constellation) [15], Aldy [16], and Stargazer [17] were used to call *CYP2D6* variants, including SVs. Using downloaded genetic reference data, we compared the *CYP2D6* variant calls of these three tools to the GeT-RM 2019 consensus genotypes [21]. As shown in Table 2, of 21 samples Stargazer called 11, Astrolabe 12, and Aldy 19 correctly (note that for NA18524 Aldy detected all existing star allels but not in the right diplotype). The number of incorrect calls seems to be linked to samples with more than two star alleles. In the sample NA18519, Aldy and Stargazer detected the *106 star allele listed as *1 in the GeT-RM 2019 consensus genotype, which we confirmed as *106 by manual evalaution of the BAM file using the Integrative Genomics Viewer [22]. As Aldy reached the highest accuracy, we analyzed 547 WGS samples (primarily European descent) using this tool (Supplementary Figure S3). While the two most frequently detected *CYP2D6* star alleles (*1, 351 alleles; *2, 187 alleles) encode a normally functioning enzyme, the most frequently detected alleles with decreased (*41, 93 alleles; *10, 25 alleles) or no function (*4, 123 alleles; *68+4, 71 alleles; *5, 30 alleles) together amount to approximately 30% of the *CYP2D6* star alleles in our cohort.

The three calling tools are comparable regarding hardware requirements, runtime, and disk footprint of output files, albeit Stargazer requires a GATK-DepthOfCoverage format file (Supplementary Figure S4). Aldy and Stargazer accept WGS, WES, and targeted sequencing data as input (cf. WGS offering best accuracy), while Astrolabe requires WGS data (i.e., unsuitable for WES).

## 3. Discussion

In this work, we outline our first-of-its-kind approach describing seamless integration of PGx profiling into our WGS-based diagnostic pipeline for rare Mendelian disorders, without generating significant additional costs. By analyzing our in-house WGS cohort as well as the largest publicly available population-based dataset gnomAD, we show that variants with DPWG recommendations comprise the vast majority of detected pharmacogene alleles and that individuals harbor at least one pharmacogenetically-actionable variant. Moreover, our results show that even the highly polymorphic, pseudogene-burdened pharmacogene *CYP2D6* may be accurately genotyped using short-read WGS, indicating that it is suitable for both diagnostics and PGx profiling in a single assay. Several discussion points and conclusions emerge from our results.

First, once a diagnosis is provided, the next step for adequate disease management ideally is a targeted medical therapy, tailored to the individual’s PGx predispositions, if available. In an optimal scenario, information regarding PGx predisposition is available prior to medical therapy. With the advent of NGS in the form of WES and WGS, unprecedented amounts of genetic data are being generated, and primarily WGS represents an often-untapped data source, as an individual’s complete PGx profile may be assessed while generating minimal additional costs. Due to decreasing sequencing costs, WGS is not prohibitively expensive for routine application and 60× WGS represents the superior solution (compared to 30× WGS and WES, Supplementary Figure S2) not only for diagnostics but also for PGx testing [14,23]. We substantiate this notion by showing that several known intronic or promoter DPWG variants (CYP2C19*17, CYP3A5*3, UGT1A1*28/*37, VKORC1*2) are not covered in gnomAD exomes due to insufficient WES capturing (depending on the used WES capturing kit), and thus incomplete treatment recommendations may be provided based on WES. Using one of several existing NGS panels, including PGRNseq [7], AmpliSeq Pharmacogenomics Research Panel (thermofisher.com), or VeraCode ADME Core Panel (illumina.com), these variants indeed would be interrogated, but at the cost of requiring an additional test. Considering that every individual on average harbors six pharmacogenetically actionable variants, we identified several patients, who require significant PGx-based dosage adjustments, which has been shown to optimize treatment outcome for instance for the anticoagulants phenprocoumon or warfarin [24].

Second, the recent technological advances have caused a paradigm shift and, currently, interpretation of (pharmaco)genetic data represents the greater challenge than their generation. In order to streamline analysis and facilitate interpretation, most providers resort to genotyping of a number of variants, of which effects on phenotype are established and translated into clear clinical guidelines. The caveat of this approach is that only the small subset of variants in the genes are interrogated, potentially missing yet undescribed or rare variants, resulting in incorrect drug recommendations. Indeed, a rare/novel LOF variant occurring in *cis* with an ultrarapid-metabolizer variant would lead to misclassification as ultrarapid metabolizer and hence lead to incorrect PGx recommendations. To estimate such cases, in gnomAD and our in-house WGS cohort we assessed the relative allele frequencies of DPWG and LOF/missense variants highly likely to disrupt the function of DPWG pharmacogenes. Thereby, we showed that the 45 analyzed DPWG variants, when combined, indeed comprise ~98% of detected pharmacogene alleles, confirming and expanding the results of two recent studies analyzing the much smaller datasets of the 1000 Genomes Project, Exome Sequencing Project, and ExAC [13,25]. As expected, the vast majority of novel LOF/missense variants occurred with a MAF <0.1%, or even with an allele count of 1. Thus, the screening of DPWG variants is of high clinical utility and cost efficient, may even be performed for individuals assessed in large-scale biobanks [19], but is ultimately limited in its scope. With increasing sequencing data available, future efforts are warranted to focus on sequencing-based approaches to enable better precision medicine, however, thereby generating novel challenges such as the interpretation of variants of unknown significance (VUS) in pharmacogenes. In the event of additional PGx variants being described, we may re-analyze our WGS data and subsequently integrate such variants into the patients’ updated PGx profiles, which are accessible using the QR code on the MSC.

Third, short-read sequencing is inherently limited in repetitive and/or homologous genomic regions (i.e., mappability <1; the so-called “dead zone” of the genome) [14], hampering accurate variant calling, for example in CYP genes. The most prominent example is the *CYP2D6* gene, responsible for bioactivation or elimination of ~25% of prescribed drugs [26], and its adjacent pseudogenes *CYP2D7* and *CYP2D8P*. Several of the 139 star alleles currently listed in PharmVar (pharmvar.org/gene/CYP2D6) are possible to detect, however, the number of duplications and gene rearrangements leading to *CYP2D6-CYP2D7* hybrid genes are notoriously difficult to genotype using short-read-based NGS approaches. To resolve these complex genotypes from short-read NGS data, freely available software tools, such as used in this study and others [18], have been introduced. Notwithstanding the powerful algorithms behind the tested tools Astrolabe, Aldy, and Stargazer, none of them assigned the correct GeT-RM 2019 consensus genotypes (generated using a variety of orthogonal methods) to all of the tested samples. In general, the tools accurately called most of the samples with simple *CYP2D6* genotypes and with only two star alleles. Discordantly called genotypes occurred in complex SVs, where Aldy reached the overall highest accuracy, not excluding the possibility that Aldy might have been trained using some of the GeT-RM samples and that the GeT-RM genotypes might be incomplete. Thus, primarily Aldy represents an important addition to the NGS software toolkit. Although incorporating bioinformatic *CYP2D6* analysis from short-read NGS data is a timesaving alternative to wetlab approaches, further evaluation of such software tools (e.g., by using long-read sequencing) is warranted. Another key challenge of PGx implementation using microarrays or short-read sequencing into actionable recommendations is phasing of variants. Several phasing algorithms exist, such as Beagle (implemented in Stargazer) [27], FastPHASE [28], and SHAPE-IT [29], all of which rely on statistical inference to assign diplotypes. The most efficient and straight forward method for variant phasing, however, is long-read sequencing, using e.g., PacBio (pacb.com) or Oxford Nanopore Technologies (nanoporetech.com). It has been shown that targeted long-read sequencing of the *CYP2D6* gene enables phasing and accurate calling of SNVs, indels, and SVs [30,31,32], which could be further evaluated using long-read WGS or hybrid assemblies of short and long reads.

Fourth, providing the PGx information as the MSC [10] or the recently developed “PGx-Passport” [33] in addition to a written report, if used correctly, allows platform-independent implementation of pharmacogenetics and patient autonomy. In theory, by integrating information on individual PGx profiles for decision support of drug selection and dosage, ADE, costs, and time can be reduced, the extent of which is currently being investigated in the PREPARE trial [9]. The Netherlands takes on a pioneering role in the implementation of pharmacogenetics, integrating PGx information in the Dutch G-Standard database, which is used by all parties in the healthcare system and provides the PGx recommendation text if a drug is prescribed or purchased [34].

Fifth, not only healthcare systems, but also the drug development industry may benefit from the implementation of PGx profiling. Currently, only 10% of all drugs that initiate phase I clinical trials are subsequently approved [2], among other reasons potentially due to neglected patient stratification such as according to metabolizer status in clinical trials. This caveat is exemplified by the angiotensin-II-type-1-receptor-antagonist losartan, which showed highly promising results in preclinical studies [35], which, however, could not be replicated in large-scale clinical trials for Marfan syndrome [36]. Because ~14% of losartan is oxidized by CYP2C9 into its 10-40× more potent metabolite E-3174, it is assumed that the majority of the effect of losartan stems from E-3174 [37]. For individuals with the frequently occurring slow metabolizer alleles CYP2C9*2,*3,*5, therefore an increased dosage of losartan might be necessary to achieve a therapeutic effect, which ultimately might explain the discrepancies between preclinical and (non-pharmacogenetically-stratified) clinical trials with losartan. Indeed, clinical trials might benefit from increased stratification according to PGx status, similarly as stratification is more common in oncology trials according to tumor driver variants [6].

We acknowledge following limitations of our study: The main limitation of our study is that for gnomAD, individual-level information is (yet) unavailable and thus we could not screen for co-occurring PGx variants in the same individual. In addition, because the here analyzed sequencing data were generated using short-read NGS, information on variant phasing is unavailable and therefore, to infer wildtype (*1) alleles, we assumed that variants detected in the same gene occur in *trans*, likely leading to an underestimation of wildtype alleles. Finally, as we limited our analysis to DPWG variants and LOF/missense variants, we likely missed other, pharmacogenetically-relevant variants, which, however, are difficult to interpret with the current knowledge. Considering these limitations, our calculations should be regarded as estimates, aiming to show the frequency of variant alleles in large-scale datasets.

## 4. Materials and Methods

### 4.1. PGx Profiling from WGS Data as well as Comparison of WGS and WES for PGx Profiling

WGS (PCR-free, 60× 150PE) of 547 individuals with rare, mainly cardiovascular or connective tissue disorders was performed as previously described [23]. Resulting raw data in the form of FASTQ files were aligned, generating binary alignment map (BAM) files, and variant calling of SNVs and small indels, generating variant call format (VCF) files, was performed using GENALICE MAP v2.5.6 (Genalice, Nijkerk, The Netherlands) as previously described [38]. For 11 DPWG pharmacogenes (*CYP2B6*, *CYP2C9*, *CYP2C19*, *CYP2D6*, *CYP3A5*, *DPYD*, *F5*, *SLCO1B1*, *TPMT*, *UGT1A1*, *VKORC1)* and an HLA-B*5701 tagging variant, common/known variants (i.e., known to generate DPWG guidelines) as well as rare/novel but potentially relevant LOF variants (SNVs and indels) were extracted from our 547 in-house genomes using GENALICE MAP’s gaVariant module, generating small gVCF-like VCF files displaying homozygous reference alleles as well (Supplementary Table S2). Note that other variant calling tools such as GATK [39] may also be used to extract pharmacogenetically-relevant variants and to generate small gVCF-like VCF files. To generate a personalized PGx report and MSC according to the DPWG guidelines, SNVs, indels, and possible SVs (such as affecting *CYP2D6*) were combined into a single VCF file and used as input for the Genetic Information Management System (GIMS) portal of bio.logis (bio.logis Genomic Healthcare GmbH, FFM, Germany).

Moreover, for the 45 DPWG variants, all the coding exons in the 11 current DPWG genes, and the core and extended ADME genes listed in pharmaadme.org, we compared the read-depth coverage of 60× and 30× WGS (TruSeq DNA PCR-Free; Illumina Inc., San Diego, CA, USA) as well as 100× WES (SureSelect Human all Exon v6 and v7; Agilent Technologies Inc., Santa Clara, CA, USA) considering the PGx profiling of 5 samples each (see Supplementary Methods and Supplementary Figure S2) [40].

### 4.2. Analysis of Star Alleles and Loss-of-Function Variants in gnomAD and in Our In-House WGS Cohort

The corresponding VCF files of gnomAD exomes v2.1.1 (125′748 exomes, released 2017, genome build GRCh37/hg19) and gnomAD genomes v3 (71′702 genomes, released 2019, genome build GRCh38/hg38) were downloaded (gnomAD.broadinstitute.com/downloads; note that as the genomes of gnomAD v2.1.1 are largely incorporated in the genomes of gnomAD v3, we considered only gnomAD v3 for genomes). In our cohort containing 547 WGS samples, joint variant calling was performed using the Population Calling module of GENALICE MAP [38] to simultaneously extract all sequence variants in 11 DPWG pharmacogenes and an HLA-B*5701 tagging variant, generating a multi-sample VCF file. Note that due to short-read-related alignment ambiguities, LOF and missense variants in *HLA-B* were not considered and the *HCP5* variant rs2395029 (Chr6(GRCh37):g.31431780T>G) was used as marker linked to the HLA-B*5701 allele in populations with European ancestry [41].

The conversion of gnomAD v3 genomic positions from GRCh38/hg38 to GRCh37/hg19 (i.e., liftover) as well as the subsequent annotation and filtering of VCF files derived from gnomAD (v2.1.1 and v3) and our in-house WGS cohort was performed using VarSeq v2.2.0 (Golden Helix Inc., Bozeman, MT, USA). To restrict the analysis to high-confidence variant calls, we excluded sequence variants with a non-PASS gnomAD filter and, as 150-bp-long reads were used, such with 150-mer mappability <1 (calculated using GEM version GEM-binaries-Linux-x86_64-20100419-003425, with m = 2) [14,42].

To determine and compare relative allele frequencies of pharmacogenetically-relevant variants in gnomAD exomes v2.1.1, genomes v3, and our in-house cohort, we applied multiple filtering criteria. Using VarSeq’s automated filter functions, we filtered for the 45 SNVs and indels implemented in the PREPARE trial [9] (i.e., the most well described/known PGx variants with DPWG guidelines) as well as for variants potentially affecting gene function: (i) Loss-of-function (LOF) variants, defined as premature termination codons (PTCs) caused by nonsense and frameshift mutations with or without expected nonsense-mediated mRNA decay, as well as canonical splice site variants (intronic ±1–2 bp) caused by single nucleotide changes and *in silico* predicted to alter splicing; (ii) missense variants classified as “damaging” or “deleterious” by all six corresponding in silico prediction tools (FATHMM, FATHMM-MKL, MutationAssessor, MutationTaster, Polyphen2, SIFT) as previously described [43]. The resulting lists of LOF and missense variants were subsequently filtered for variants listed in ClinVar (v2019.11; ncbi.nlm.nih.gov/clinvar) as “Drug Response” [44] and/or in the Human Gene Mutation Database (HGMD) professional (v2019.4; portal.biobase-international.com) as “FP”, “DFP”, “DM?”, or “DM” in the context of drug response [45], and/or the PharmGKB database (v2019.6; pharmgkb.org/downloads) [46].

For all 11 analyzed pharmacogenes, relative allele frequencies of DPWG and LOF variants were assessed and compared among gnomAD v2.1.1, v3, and our in-house WGS cohort. To infer the number of wildtype (*1) alleles, we subtracted the number of detected DPWG and LOF alleles from the total allele numbers in gnomAD v3, v2.1.1, and our in-house cohort under the assumption that variants detected in the same gene occur in *trans*. In addition, in our in-house WGS cohort, but not in gnomAD, we were able to assess the co-occurrences of DPWG, LOF and/or likely pathogenic missense variants in the same individual.

### 4.3. Evaluation of CYP2D6 Variant Callers

For the SV detection in *CYP2D6*, we evaluated the software tools Astrolabe (previously Constellation) v0.8.6.1 [15], Aldy v2.2.3 [16], and Stargazer v1.0.7 [17]. To compare the accuracy of these three variant callers, we downloaded FASTQ and/or BAM files of 20 human PGx reference WGS samples from the European Nucleotide Archive (ENA, ebi.ac.uk) (HG00436, NA07029, NA18959, NA19109, NA21781, NA12873, NA18861, HG00589, NA19917, NA07019, NA12717, HG00276, NA18524, NA18540, NA07348, NA18519; NA18966, NA18992 and NA19226) [47] or from ftp-trace.ncbi.nih.gov/1000genomes/ftp/phase3/data/NA12892 (NA12892). Variant calling was performed using GENALICE MAP [38] for all but one (NA12892) of these downloaded samples. For NA12892, we used Strelka2 v2.9.10 [48] because only the BAM file was available for download (cf. GENALICE MAP requires FASTQ file as input). In addition, for the reference sample NA12878 (termed NA12878 in-house), we analyzed the genomic DNA by means of our in-house WGS pipeline (PCR-free, 60× PE150) using Isaac v01.15.02.08 for alignment and v2.0.13 for variant calling [49]. For the software tool Aldy, only BAM files were required as inputs, while the tool Astrolabe needed VCF and BAM files. For Stargazer, in addition to BAM files, GATK-DepthOfCoverage format (GDF) files were required as input (generated using GATK v3.5) and *RYR1* served as control gene. All three SV detection tools were applied using default settings (Supplementary Figure S4). For each reference sample, the results of SV calling were compared to the consensus *CYP2D6* genotype obtained from the GeT-RM projects 2019 [21]. For resolving ambiguous calls, BAM files were manually evaluated using the Integrative Genomics Viewer v2.4.19 [22]. Moreover, we used Aldy [16] to assess the frequencies of *CYP2D6* star alleles in our in-house WGS cohort.

### 4.4. Statistical Analysis

The upper and lower limits of 95% confidence intervals of relative frequencies were calculated using the online tool VassarStats with a correction for continuity (vassarstats.net/prop1.html).

## 5. Conclusions

Taken together, we show that short-read WGS, rather than WES, is suitable for the profiling of pharmacogenes, including *CYP2D6*. WGS-based clinical sequencing may therefore be the most comprehensive all-in-one approach for the simultaneous testing of Mendelian diseases and profiling of pharmacogenes without generating significant additional costs. Moreover, we demonstrate that known DPWG variants comprise the majority of PGx variation. Hence, restricting PGx profiling to these variants streamlines the interpretation process and provides appropriate pharmacogenetically-guided treatment recommendations for the vast majority of individuals. For true precision medicine, i.e., for the best possible pharmacogenetically-guided treatment recommendations for each patient, however, it is warranted that comprehensive approaches, such as presented here, expand the targeted profiling of known DPWG variants to the genome-wide profiling of all pharmacogenes.

## Figures and Tables

**Table 1:** Number (n) and proportions (%) of DPWG pharmacogene variants detected in gnomAD and our in-house WGS cohort.

Cohort	gnomAD Exomes v2.1.1		gnomAD Genomes v3		In-House WGS Cohort	
Cohort Size	125’748 Exomes		71’702 Genomes		547 Genomes	
**Novel LOF/missense variants** (not in ClinVar/HGMD/PharmGKB) ^1^	**n**	**%**	**n**	**%**	**n**	**%**
Total variants (alleles)	823 (4′214)	100.0	512 (6′940)	100.0	10 (10)	100.0
MAF > 5%	0	0.0	0	0.0	0	0.0
0.1% < MAF < 5%	2	0.2	8	1.6	0	0.0
MAF < 0.1%	821	99.8	504	98.4	10	100.0
**Known LOF/missense variants** (in ClinVar/HGMD/PharmGKB) ^1^	**n**	**%**	**n**	**%**	**n**	**%**
Total variants (alleles)	103 (5′584)	100.0	80 (2′706)	100.0	4 (17)	100.0
MAF > 5%	0	0.0	0	0.0	0	0.0
0.1% < MAF < 5%	5	4.9	3	3.7	1	25.0
MAF < 0.1%	98	95.1	77	96.3	3	75.0
**DPWG variants**	**n**	**%**	**n**	**%**	**n**	**%**
Total variants (alleles)	40 (375′331)	100.0	45 (487′758)	100.0	37 (4′162)	100.0
MAF > 5%	10	25.0	14	31.1	14	37.8
0.1% < MAF < 5%	22	55.0	23	51.1	18	48.7
MAF < 0.1%	8	20.0	8	17.8	5	13.5

^1^ LOF/missense variants in *HLA-B* were not considered due to short-read-related alignment ambiguities. Abbreviations: DPWG, Dutch Pharmacogenetics Working Group; LOF, loss of function; MAF, minor allele frequency.

**Table 2:** Comparison of *CYP2D6* callers output, in form of star alleles (single nucleotide variants, small insertions/deletions, structural variations), from 21 publicly available short-read WGS samples.

Reference Samples	GeT-RM Consensus Genotype 2019	Astrolabe v0.8.6.1	Aldy v2.2.3	Stargazer ^1^ v1.0.7
HG00436	*2×2/*71	*2/*71	*2×2/*71	*1/*83+*2
NA07029	*1/*35	*1/*35	*1/*35	*1/*35
NA18959	*2/*36+*10	*2/*10	*2/*36+*10	*2/*36+*10
NA19109	*2×2/*29	*2/*29	*2×2/*29	*29/*83+*2
NA21781	*2×2/*68+*4	*2/*4 ^2^	*2×2/*68+*4	*4N+*4/*68+*4
NA12878 in-house	*3/(*68)+*4	*3/*4 ^3^	*3/*68+*4	*3/*4
NA12873	*1/*5	*1/*5	*5/*61 ^4^	*1/*5
NA18861	*5/*29	*5/*29	*5/*29	*13C/*29
HG00589	*1/*21	*1/*2	*1/*21	*1/*2
NA19917	*1/*40	*1/*40	*1/*40	*1/*40
NA07019	*1/*4	*1/*4	*1/*4	*1/*4
NA12717	*1/*1	*1/*1	*1/*1	*1/*1
HG00276	*4/*5	*4/*4	*4/*5	*4/*5
NA18524	*1/*36×2+*10	*1/*10	*1+*36/*36+*10 ^5^	*1/*10×3
NA18540	(*36+)*10/*41	*41/*10 ^3^	*36+10/*61+*69	*10×2/*41×2
NA12892	*2/*3	*2/*3	*2/*3	*2/*3
NA07348	*1/*6	*1/*6	*1/*6	*1/*6
NA18519	*1/*29 ^6^	*1/*29	*106/*29	*106/*29
NA18966	*1/*2	*1/*2	*1/*2	*1/*2
NA18992	*1/*5	*1/*5	*1/*5	*1/*13C
NA19226	*2/*2×2	*2/*2 ^2^	*2/*2×2	*2/*83+*2
**Total true**		**12**	**19**	**11**
**Total false**		**9**	**2**	**10**

^1^ Stargazer outputs additional possible star alleles, but only the main star alleles are shown in this table. ^2^ Possible gene duplication detected by Astrolabe. ^3^ Possible gene hybrid *68 detected by Astrolabe. ^4^ Aldy could not phase any major solution, only potential star (*) alleles. ^5^ Aldy detected the existing variants, although not in the same diplotype as in the GeT-RM consensus genotype 2019, and thus we counted the call as correct. ^6^ The allele *106 is not listed in the GeT-RM consensus genotype, but detected by Stargazer and Aldy and manually confirmed using the Integrative Genomics Viewer. Green and red colors denote correct and incorrect calls, respectively. SNVs and indels are denoted by *2, *3, *4, *6, *10, *21, *29, *35, *36, *40, *41, *69, *71, *83, *106, and SVs by *4N, *5, *13, *61, *68.

## Supplementary Materials

Supplementary materials can be found at https://www.mdpi.com/1422-0067/21/7/2308/s1.
