# Supplementary material for: Added Value of Clinical Sequencing: WGS-Based Profiling of Pharmacogenes
Source: Int J Mol Sci. 2020 Mar 26;21(7):2308. doi: 10.3390/ijms21072308 (PMC7178228; doi:10.3390/ijms21072308)
Supplement: Supplementary file 1 [file ijms-21-02308-s001.zip › Caspar_et_al_Supplementary_Material.doc]

Supplementary Material

**Added Value of Clinical Sequencing: WGS-Based Profiling of Pharmacogenes**

**Sylvan M. Caspar ^1,2^, Timo Schneider ^1^ , Janine Meienberg ^1^ and Gabor Matyas ^1,3,^***

^1^	Center for Cardiovascular Genetics and Gene Diagnostics; Foundation for People with Rare Diseases, 8952 Schlieren-Zurich, Switzerland; caspar@genetikzentrum.ch (S.C.); meienberg@genetikzentrum.ch (J.M.);

^2^	Laboratory of Translational Nutrition Biology, Department of Health Sciences and Technology, ETH Zurich, 8603 Schwerzenbach, Switzerland;

^3^	Zurich Center for Integrative Human Physiology, University of Zurich, 8057 Zurich, Switzerland

*****	Correspondence: matyas@genetikzentrum.ch; +41 43 433 86 86

Received: date; Accepted: date; Published: date

**Supplementary Methods**

We compared whole-genome sequencing (WGS, native 60× and downscaled 30×; Illumina TruSeq DNA PCR-Free; sequenced on an Illumina HiSeq X Ten; Illumina Inc., San Diego, CA, USA) and whole-exome sequencing (WES; native 100×) using the whole-exome capture platforms Agilent (Agilent Technologies Inc., Santa Clara, CA, USA) SureSelect Human all Exon v7 (sequenced on an Illumina NovaSeq 5000) and v6 (sequenced on an Illumina HiSeq X Ten) regarding their coverage performance for pharmacogenetic profiling. We considered five samples for each technique as well as performed alignment (GRCh37/hg19) and variant calling using GENALICE MAP [1] for all samples. As previously described [2], we calculated and compared the read coverage from the generated BAM files (excluding mapping quality 0) using SeqMonk v1.39.0 (bioinformatics.babraham.ac.uk/projects/seqmonk) for the 45 DPWG variants, the coding exons in the 11 current DPWG genes, and an extended set of PGx genes, referred to as “Pharmaadme Genes” including the core and extended ADME genes listed in pharmaadme.org in addition to the 11 current DPWG genes (Supplementary Figure S2). Coding regions were defined according to the Table “RefSeq all” in the track “NCBI RefSeq” from the UCSC Table browser (genome.ucsc.edu/cgi-bin/hgTables). Genes with frequent copy number loss and such located on chromosome Y were excluded from the analysis. Exons/variants were defined as incompletely covered if at least one position was covered <21×. In addition, we performed our read-coverage calculations restricted to genes, exons or variants having a complete 150-mer mappability =1 [3,4].

**Supplementary Table S1.** Overview of detected sequence variants (see separate multi-sheet Excel table).

**Supplementary Table S2.** Overview of sequence variants, which currently are implemented in our PGx-profiling pipeline (see separate Excel table).

**Supplementary Figure S1.** Distribution of the number of detected sequence variants with current treatment recommendations according to the Dutch Pharmacogenetics Working Group (DPWG) guidelines, based on our in-house WGS cohort of 547 genomes.

**Supplementary Figure S2.** Read coverage calculations for whole-genome sequencing (WGS, 60× and 30×) and whole-exome sequencing (WES, Agilent SureSelect Human All Exon v6 and v7). Bars represent the percentage of completely covered (i.e. ≥21× at each nucleotide position) genes, exons or DPWG variants, as means of five samples each (error bars indicate 95 % confidence intervals). “Pharmaadme Genes” (n = 286) contains all core and extended pharmacogenetically-relevant genes listed in pharmaadme.org as well as the 11 DPWG genes (*CYP2B6*, *CYP2C9*, *CYP2C19*, *CYP2D6*, *CYP3A5*, *DPYD*, *F5*, *SLCO1B1*, *TPMT*, *UGT1A1*, *VKORC1*).

**Supplementary Figure S3.** Frequency of *CYP2D6* star alleles detected by the software tool Aldy in our in-house cohort of 547 short-read WGS (60× PE150, PCR-free) samples.

**Supplementary Figure S4.** Overview of (**a**) used whole-genome sequencing samples and (**b**) required inputs for the compared *CYP2D6* variant callers. Abbreviations: BAM: binary alignment map; GDF: GATK-DepthOfCoverage format; VCF: variant call format.

Supplementary References

1. Plüss, M.; Kopps, A.M.; Keller, I.; Meienberg, J.; Caspar, S.M.; Dubacher, N.; Bruggmann, R.; Vogel, M.; Matyas, G. Need for speed in accurate whole-genome data analysis: GENALICE MAP challenges BWA/GATK more than PEMapper/PECaller and Isaac. *Proc*. *Natl*. *Acad*. *Sci*. *USA* **2017**, *114*, E8320–E8322, doi:10.1073/pnas.1713830114.
2. Meienberg, M.; Zerjavic, K.; Keller, I.; Okoniewski, M.; Patrignani, A.; Ludin, K.; Xu, Z.; Steinmann, B.; Röthlisberger, B.; Schlapbach, R.; et al. New insights into the performance of human whole-exome capture platforms. *Nucleic Acids Res*. **2015**, *43*, e45, doi:10.1093/nar/gkv216.
3. Caspar, S.M.; Dubacher, N.; Kopps, A.M.; Meienberg, J.; Henggeler, C.; Matyas, G. Clinical sequencing: From raw data to diagnosis with lifetime value. *Clin*. *Genet*. **2018**, *93*, 508–519, doi:10.1111/cge.13190.
4. Derrien, T.; Estellé, J.; Marco Sola, S.; Knowles, D.G.; Raineri, E.; Guigó, R.; Ribeca, P. Fast computation and applications of genome mappability. *PLoS ONE* **2012**, *7*, e30377, doi:10.1371/journal.pone.0030377.
